# Ketogenic diet therapy for the treatment of pediatric epilepsy

**DOI:** 10.1002/epd2.20320

**Published:** 2024-12-12

**Authors:** Babitha Haridas, Alexander Testino, Eric H. Kossoff

**Affiliations:** ^1^ Department of Neurology and Pediatrics Johns Hopkins Hospital Baltimore Maryland USA

**Keywords:** epilepsy, ketogenic diet, ketogenic diet therapy, modified Atkins diet, refractory epilepsy, seizures

## Abstract

In 1921, the classic ketogenic diet was created at the Mayo Clinic in Rochester, Minnesota to treat epilepsy in children and adults. Over a century later, it is a widely used, standard‐of‐care therapy for typically treatment‐resistant epilepsy worldwide. There are currently five versions of ketogenic diet therapy that can be started either in or out of the hospital setting. It is overall effective in approximately half of children started, usually within a few months. Established indications for ketogenic diet therapy exist, in which this treatment may potentially even be more advantageous than antiseizure medications. Some of these indications include Glut1 deficiency, pyruvate dehydrogenase deficiency, infantile epileptic spasms syndrome, epilepsy with myoclonic‐atonic seizures, and formula‐fed children. Although most children are also receiving antiseizure medications with ketogenic diet therapy, its use may lead to medication reduction or withdrawal in some cases, and improvement in cognition and quality of life. Supplements are begun when ketogenic diet therapy is initiated in order to prevent common side effects, including constipation, kidney stones, growth disturbance, and dyslipidemia. Typically, after 2 years in most children, ketogenic diet therapy is discontinued gradually.

**Key points**

- The ketogenic diet is a nonpharmacologic therapy for the treatment of pediatric epilepsy with more than 100 years of usage.
- There are five ketogenic diets: classic ketogenic diet, modified Atkins diet, medium‐chain triglyceride diet, modified ketogenic diet (UK), and low glycemic index treatment.
- Indications exist and include several epilepsy syndromes including Glut1 deficiency syndrome, Dravet syndrome, epilepsy with myoclonic‐atonic seizures, infantile spasms and more.
- Diet therapy is being increasingly used for super‐refractory status epilepticus.
- Ketogenic diets need to be started with a neurologist and dietitian's supervision.
- Initiation can be done as an inpatient or outpatient, with or without a fasting period, quickly or slowly.
- Supplements including vitamins, calcium, and minerals can prevent adverse effects.
- Side effects exist including constipation, gastroesophageal reflux, kidney stones, dyslipidemia, and growth disturbance.
- Diet therapy is used for at least 3 months ideally, and often discontinued after 2 years.
- Diet therapy can be retried a second time after years if clinically appropriate.

## 1 ILAE LEARNING OBJECTIVE

### 1.1 Demonstrate knowledge of indications, limitations, and risks for ketogenic diet

- The reader will understand the current list of indications (and contraindications) for ketogenic diet therapy (KDT) from the 2018 consensus guideline.
- The reader will realize the situations in which KDT could be used first‐line or in an emergency situation.
- The reader will be able to list the common and rare side effects of KDT.

### 1.2 What is the ketogenic diet?

The classic ketogenic diet was created at the Mayo Clinic in 1921 as a form of antiseizure therapy in children and adults. Today, the ketogenic diet is one of the four major pillars of antiseizure therapies along with antiseizure medications, epilepsy surgery, and neuromodulation. While the classic ketogenic diet is effective, to maximize tolerability several “alternative” dietary therapies have been formulated, including the medium‐chain triglyceride (MCT) diet, low glycemic index treatment (LGIT), modified ketogenic diet (UK), and modified Atkins diet (MAD).

#### 1.2.1 Classic ketogenic diet

The classic ketogenic diet is a high fat, adequate protein, low carbohydrate diet. The diet is implemented using a specific ratio of fats to carbohydrates and proteins combined. A 4:1 ratio is typically used in most cases.^1^ A lower ketogenic ratio of 3:1 is used among infants, adolescents, and to assist with improved tolerability.^1^ In a randomized control trial of 76 children with drug resistant epilepsy, the efficacy and tolerability of a classic 4:1 diet was compared to 3:1 diet. Fifty‐five percent of children on the 4:1 ratio were seizure free, in comparison with 30.5% of children on 3:1 ratio (*p* < .05).^2^ Additionally, 10/12 (83%) of patients who were not seizure free on a ratio of 3:1 showed increased seizure reduction after changing to 4:1 ratio.^2^ The diet was better tolerated in children on 3:1 as compared to 4:1 diet with 35% on a 4:1 KD reporting gastrointestinal intolerance, as opposed to 14% on 3:1.^2^

#### 1.2.2 Medium‐chain triglyceride diet

The MCT diet is an alternative form of ketogenic dietary therapy created in the 1970s. While the main source of fat in the classic ketogenic diet is long‐chain triglycerides, the MCT diet utilizes more medium‐chain triglycerides. Since MCTs provide more ketones per kilocalorie of energy in comparison with long‐chain triglycerides, this permits an increase in the amount of carbohydrates, thus improving tolerability of a ketogenic dietary therapy. In a randomized control trial including 145 children with drug resistant epilepsy to receive either the MCT or CKD, there was no significant differences between the groups in numbers achieving greater than 50% or 90% seizure reduction.^3^ The MCT diet is more commonly used in Canada and the United Kingdom, but other centers have implemented it as a way to allow for more carbohydrates. MCT fats may have advantages in regard to less risk of hypercholesterolemia and constipation (as they can be laxative).

#### 1.2.3 Low glycemic index treatment

The LGIT is an alternate version to the classic ketogenic diet, created at the Massachusetts General Hospital to optimize tolerability by permitting the use of more carbohydrates, with the permitted carbohydrates having a low glycemic index (typically <50). Children on this diet have very low to absent levels of ketosis. Among 76 patients who were on LGIT, greater than 50% seizure reduction was noted in 54% and 66% of patients at 6 and 12 months, respectively.^4^ Notably, only three patients reported side effects of transient lethargy in this cohort.^4^ It has been reported as highly effective particularly for children with Angelman syndrome.^5^

#### 1.2.4 Modified Atkins diet

This form of ketogenic diet therapy has been in use for over two decades and was created by our center (Johns Hopkins Hospital). While there are many similarities with the Atkins diet, the term “modified” was intentionally included in the title of this therapy at its inception in 2006 to distinguish it from the Atkins diet in regard to the lower carbohydrate limit (10–20 g/day), high fat intake, and the goal of seizure control (as opposed to weight loss).^6^ MAD is a high fat, low carbohydrate form of dietary therapy that provides approximately a 1:1 or 2:1 ketogenic ratio.^1^ Due to significant day to day variability, a ketogenic ratio is not tabulated in patients on this diet and foods are not calculated and measured using a gram scale as with the classic ketogenic diet. Carbohydrates are restricted to 20 grams/day, proteins are not measured, and fats are strongly encouraged.^7^ Early efficacy trials showed 65% patients had >50% seizure reduction, with 35% having >90% seizure reduction.^8^ Randomized controlled trials comparing MAD with antiseizure medications found >50% seizure reduction in 52% of patients on MAD, in comparison with 11.5% on medications.^9^ Sharma et al. also conducted a study “simplifying” the MAD for use among families with a low literacy level in India.^10^ The process of “simplification” included transitioning from using a gram scale to using standardized measuring equipment and changes in recipe designs. In this cohort, there was >50% seizure reduction in 56% of patients, compared to 7.5% with ongoing antiseizure medication therapy.^10^ The ability to start this diet as an outpatient, relative flexibility, and fewer adverse effects make this a potential diet therapy of choice for adolescents and adults.^6^

#### 1.2.5 Modified ketogenic diet (UK)

MKD is the most recent alternative diet, utilizing many of the principles of the MAD but using weighed portions and household measures to calculate a diet prescription.^11^ With MKD, fats provide approximately 75% of energy, protein 20%, while carbohydrates account for 5% (approximately 15‐30 g). MKD can be initiated without a fasting period and can be started as an outpatient, similar to the MAD and LGIT.^11^ To date, there are no specific efficacy studies for this diet therapy.

## 2 PATIENT SELECTION

Ketogenic diet therapies can effectively treat epilepsy in patients from infancy through adulthood. The ketogenic diet was initially avoided in children under 2 years of age for many decades, in view of this time period being crucial to development, infants not being thought to achieve ketosis, and perceived high risk of nutritional inadequacies.^12^ However several studies have revealed that it is safe to use in this cohort.^12^, ^13^, ^14^ Adolescents and adults can also go on ketogenic diet therapy, typically with the MAD, MKD, or LGIT.^15^, ^16^ The diet is effective for all seizure types and genders as well. In the next section, we will discuss how specific epilepsy syndromes and situations may predict a better outcome to KDT.

## 3 INDICATIONS AND CONTRAINDICATIONS

There are several conditions for which KDT have been consistently reported as producing 60–70% responder rates, higher than the typical 50% responder rate overall seen in children after 6 months. These conditions have been classified as “indications” by the International Ketogenic Diet Study Group (Table 1, Kossoff 2018).^1^

**TABLE 1:** Epilepsy syndromes and conditions for which KDT has been consistently reported as more beneficial (>70%) than the average 50% KDT response (defined as >50% seizure reduction).^1^

Angelman Syndrome
Complex 1 mitochondrial disorders
Dravet syndrome
Epilepsy with myoclonic‐atonic seizures (Doose syndrome)
Febrile infection‐related epilepsy syndromes (FIRES)
Formula‐fed (solely) children or infants
Glucose transporter protein 1 (Glut1) deficiency syndrome
Infantile Epileptic Spasms Syndrome
Ohtahara syndrome
Pyruvate dehydrogenase deficiency
Super‐refractory status epilepticus
Tuberous sclerosis complex

### 3.1 Angelman syndrome

Angelman syndrome is a neurodevelopmental disorder characterized by severe developmental delay, speech impairment, uncontrolled laughter, and ataxia.^17^ Epilepsy occurs in up to 90% of patients. There are no comparative trials of various antiseizure therapies; the consensus recommendation is to treat with ASMs as first‐line therapy and to consider CKD and LGIT as dietary options.^5^ In a case series, 12/12 patients reported a decrease in seizures with LGIT, while 10/12 (83%) reported >90% reduction in seizure frequency.^18^ CKD is recommended for infants and children with gastrostomy tubes.^5^ LGIT is suggested for other children.^5^ If LGIT does not provide adequate control, it can be transitioned to CKD.^5^ In one study, 11/31 patients placed on the CKD reported it to be the best overall treatment.^19^

### 3.2 Complex 1 mitochondrial disorders

This is one of the most commonly identified biochemical mitochondrial defects and is associated with epilepsy.^20^ In patients with respiratory chain complex defects, the use of ketone bodies can result in heteroplasmic shifting between and within cells.^21^ Ketone bodies can be useful in differentiating between normal cells and respiration compromised cells; hence, KDT can be useful in heteroplasmic mitochondrial DNA disorders.^21^ Ten of 14 patients with refractory epilepsy with respiratory chain complex defects had a reduction in seizure frequency. KDT permitted 80% of patients to reduce or discontinue their ASMs.^22^

### 3.3 Dravet syndrome

Dravet syndrome is a developmental and epileptic encephalopathy characterized by multiple seizure types, recurrent status epilepticus, cognitive impairment, and developmental delays. In a multicenter retrospective observational study of 114 patients with DS on KDT, rates of seizure freedom at 3 and 6 months were 32.5% and 30.7%, respectively.^23^ A 70% responder rate was observed in patients with DS on KD. It was found to be equally effective when compared to combination therapy with stiripentol, clobazam, and valproic acid.^24^

### 3.4 Epilepsy with myoclonic‐atonic seizures

EMAtS, also referred to as Doose syndrome, typically starts in developmentally normal children between 2 and 6 years of age with an explosive “stormy” onset of multiple seizure types that are typically refractory to medications.^25^ Response to dietary therapy has been significantly greater than response to ASM. Studies have indicated an overall response to the first three ASMs being 26%, compared to KD being effective in 79%.^26^ In a retrospective review, 83% experienced >50% seizure reduction and 47% were seizure free after 2 years of the MAD.^27^

### 3.5 Febrile infection‐related epilepsy syndrome

FIRES is a subcategory of New Onset Refractory Status Epilepticus (NORSE) that can occur in all age groups. This type of refractory status epilepticus develops following a febrile illness that started between 2 weeks and 24 h before onset of RSE in an otherwise healthy patient.^28^ Due to the likely underlying immune mechanisms in sustaining seizures, the ketogenic diet and second‐line immunotherapies are initiated in non‐infectious NORSE/FIRES with inadequate response to first‐line immune treatment.^29^ Early initiation of KDT was noted to improve outcomes.^30^

### 3.6 Formula‐fed infants and children

KDT can be initiated with relative ease in formula‐fed children, particularly those with pre‐existing gastrostomy tubes. In a study of 226 patients who were on KD, 61 were formula fed. All 61 patients had better seizure control than the typical solid food fed child.^31^ Recently, a study found that using formula increased that chance to respond to treatment 7.32 times.^32^ Although the likely explanation of why this preferential improvement would be compliance, the benefits of KDT as formulas may be more complicated than that. Formula has improved the palatability of the diet, ease of calculation of components, and is cost effective as it is typically covered by insurance companies if it is the majority of nutritional needs for the patient.^31^

### 3.7 Glucose transporter type 1 deficiency syndrome

Glut‐1 is normally expressed in the endothelial cells of the blood–brain barrier. A deficiency in Glut‐1 results in reduced glucose transport across the blood–brain barrier. Clinical manifestations include early onset absence epilepsy, paroxysmal eye‐head movements, ataxia, developmental delays, and paroxysmal exertion‐induced dystonia.^33^ KDT is the treatment of choice for Glut1DS.^1^ The diet provides ketones that bypass the metabolic defect and provides an alternative cerebral fuel for the developing brain.^1^, ^33^ CKD should be used in infants and children for as long as possible.^12^, ^33^ MAD can be used in school‐age children, adolescents, and adults.^33^

### 3.8 Infantile epilepsy spasms syndrome

IESS, previously known as West syndrome, consists of the triad of infantile spasms, EEG pattern of hypsarrhythmia, and developmental delay or regression. In a meta‐analysis, 60% patients experienced >50% seizure reduction with KDT.^34^ A retrospective case–control study demonstrated 8/13 patients with IESS started on CKD as first‐line therapy were spasm free within 18 days.^35^ Those who did not respond to KD were treated with hormonal therapy.^35^ Similar results were found by a more recent study.^36^

### 3.9 Ohtahara syndrome

Ohtahara syndrome manifests with tonic spasms, focal motor seizures, refractory epilepsy, and an EEG with a burst suppression pattern.^37^ These patients have a poor prognosis and may evolve into IESS and in many cases to Lennox Gastaut Syndrome (LGS).^38^ Several publications have reported a substantial reduction in seizures with the CKD.^39^, ^40^

### 3.10 Pyruvate dehydrogenase deficiency

Patients with PDHD are unable to metabolize pyruvate to acetyl CoA, leading to increased lactate production and impaired energy production. PDHD commonly manifests with numerous neurological signs such as congenital microcephaly, hypotonia, ataxia, developmental delays, and epilepsy.^41^ Patients may have abnormal neuroimaging and metabolic abnormalities, such as lactic acidosis.^41^ KDT provides ketone bodies that act as an alternate fuel for the developing brain.^1^, ^41^ Among 19 pediatric patients with PDHD, all patients with seizures treated with KD improved during treatment. Approximately 50% had resolution of seizures within 1 year of diet initiation.^42^

### 3.11 Super‐refractory status epilepticus

Super‐refractory status epilepticus (SRSE) is defined as status epilepticus that persists at least 24 h or more after the onset of anesthetic therapy, or recurs with the reduction/withdrawal of anesthesia.^43^ Among 14 patients in a prospective multicenter study, ketosis was achieved in a median of 2 days with EEG resolution of seizures in 7 days of initiation in 71% of patients.^44^ Nearly 80% of patients were weaned off of continuous infusions 2 weeks after starting KD. This improvement persisted beyond the acute period with 7/12 patients with a 3‐month follow‐up showing continued improvement in seizures with 4/12 being seizure free.^44^ Favorable responses have been found among pediatric and adult patients. Both enteral and parenteral formulations are available. Concurrent use of propofol with KDT potentially raises the risk of propofol infusion syndrome and probably should be avoided.^45^

### 3.12 Tuberous sclerosis complex

Tuberous sclerosis complex (TSC) is a genetic multisystem disorder typically caused by mutations in TSC1 or TSC2. Epilepsy is reported in 75%–90% of patients with TSC.^46^ At 3 months after KD initiation, 83% of patients had >50% seizure reduction with nearly 60% having qualitative improvement in cognition per caregiver report.^47^ Six of 10 patients that continued the KD for 6 months were seizure free and 80% achieved >50% seizure reduction.^47^

## 4 CONTRAINDICATIONS

KDTs exert their action by shifting from the use of carbohydrates to lipids as the primary energy source. A disorder of lipid metabolism, for example, primary carnitine deficiency, may worsen with the initiation of KDT or with fasting.^1^ The IKDSG has provided the following list of absolute and relative contraindications (Table 2).^1^

**TABLE 2:** Contraindications to the use of Ketogenic Diet Therapies.^1^

Absolute Primary carnitine deficiency Carnitine palmitoyltransferase (CPT) I or II deficiency Carnitine translocase deficiency Beta‐oxidation defects Medium‐chain acyl dehydrogenase deficiency (MCAD) Long‐chain acyl dehydrogenase deficiency (LCAD) Short‐chain acyl dehydrogenase deficiency (SCAD) Long‐chain 3‐hydroxyacyl‐CoA deficiency Medium‐chain 3‐hydroxyacyl‐CoA deficiency Pyruvate carboxylase deficiency Porphyria
Relative Inability to maintain adequate nutrition Surgical focus identified by neuroimaging and video‐EEG monitoring Parent or caregiver non‐compliance Propofol concurrent use (risk of propofol infusion syndrome may be higher)

## 5 INITIATION OF DIETARY THERAPY

Despite the ketogenic diet being over 100 years old, the basics of initiation of dietary therapy had not been questioned or studied until the past 10–20 years. The classic ketogenic diet is traditionally started as an inpatient in the hospital setting over a few days while foods are gradually introduced to a child who had been fasted for 24–48 h (clear, calorie‐free fluids only). A ratio is chosen and the dietitian calculates meal plans based on that ratio and a set number of calories calculated for that individual child. Medication doses are usually left unchanged, with a change over to tablet formulations (as liquid medications have minimal, but potentially ketone reducing carbohydrates). This may not be completely necessary, however, in all patients if the ketogenic ratio is increased.^48^ In the hospital setting, foods are usually weighed and measured by the kitchen staff or a dietitian, and the family is instructed on how to do this themselves. In fact, education is a large part of the ketogenic diet initiation period. Computer programs (e.g., KetoDietCalculator™) can be very helpful for families once they are discharged.

Although tradition has incorporated both a fasting period and inpatient stay, recent studies have questioned the role and universality of this approach. Fasting, in a randomized trial, led to equal levels of blood ketones after 1 week, and equivalent seizure control outcomes after 3 months.^49^ However, fasting does lead to a quicker (in days) onset of ketosis which may be desired.^50^ In addition, many centers nowadays implement the ketogenic diet with an outpatient approach, letting the child and family remain at home during initiation. This allows the family to start preparing and cooking the foods immediately, and the child will likely be more comfortable (and eat more) at home than in the hospital. Education is done in clinics, and typically, the child will be nearby in case of hypoglycemia, vomiting, or acidosis.

Novel methods of initiating ketogenic diet therapy have emerged in recent years as well. In the inpatient setting, the ketogenic diet can be started by increasing the ratio every day (e.g., 2:1, 3:1, 4:1) versus the tradition of keeping the ratio stable and increasing calories daily.^49^ Some centers have also increased the ketogenic diet as an outpatient even more slowly (over months).^51^ Virtual ketogenic diet initiations in the recent COVID‐19 pandemic have been successful and are a valid option.^52^ Lastly, the MAD and LGIT are nearly universally started without a fast and as an outpatient as part of their design.^1^ No matter how KDT is started, it needs to be ideally implemented under the care of both a physician and nutritionist.^1^

## 6 ANTISEIZURE DRUGS AND THE KETOGENIC DIET

Although often seen as mutually exclusive therapies for epilepsy, they are typically used together, in one study up to 86% of patients.^53^ Therefore, the interactions (both positive and negative) between KDT and antiseizure drugs are very important to be aware of. This topic was covered in great detail in a recent review; we would suggest referring to this for more detail.^54^

One major secondary reason for starting KDT is to wean medications and a child does not need to be seizure‐free in order to make an attempt.^1^ If successful, this may lead to reduced treatment costs and improved alertness. Most centers will wait at least 1 month after starting KDT to try to wean antiseizure drugs, although one study reported success discontinuing medications as early as the first month.^1^, ^55^

The interactions between cannabidiol (CBD) and KDT have not been fully elucidated; however, the two therapies have similar indications including LGS and Dravet syndrome.^54^ The package insert for the commercial product, Epidiolex™, has reported that ingestion with a “high fat/high calorie” meal results in an increase in C‐max by five‐fold, as opposed to fasting state in healthy volunteers (https://pp.jazzpharma.com/pi/epidi olex.en.USPI.pdf). There have been reports of an additive effect and improved seizure control when MCT oil is used as the vehicle for CBD.^54^ However, this combination has also been associated with increased MCT‐related gastrointestinal side effects of abdominal pain, diarrhea, nausea, and vomiting.^54^ There are also anecdotal reports of higher ketosis with CBD use.^54^

Some drugs that have been reported as problematic for efficacy on KDT, and perhaps worth trying to wean early, include phenobarbital, valproate, and lamotrigine.^54^ Zonisamide and vagus nerve stimulation may work well with KDT based on studies as well.^56^, ^57^ However, the evidence for this is limited at best. It is also unclear if antiseizure medication levels decrease on KDT; dose adjustment is not necessary. Traditionally, medications are switched from liquid to tablet formulation to reduce hidden carbohydrates, but this also may not be necessary or can be accounted for with extra fat.^48^ Finally, it is acceptable to consider addition of an antiseizure medication after several months and various KDT “fine‐tuning” attempts have been tried in order to improve seizure control. In one study, this was a successful method of seizure reduction in 24% of children on KDT.^58^

## 7 SUPPLEMENTS

Supplementation with vitamins is necessary for patients on KDT due to the lack of B vitamins, calcium, and Vitamin D in typical foods provided. Children should be started on a daily multivitamin along with calcium and Vitamin D in all cases, except if a KDT formula is utilized that possesses sufficient vitamins and minerals. Additional supplementation has been advocated based on some studies, but is generally optional. These supplements include extra zinc, selenium, magnesium, laxatives, gastric acid blockers, or probiotics.^1^ Oral citrates have been demonstrated in one study to reduce the risk of kidney stones from 6.7% to .9% when used empirically and possibly may also reduce acidosis.^59^ Some centers will also add additional carnitine to help boost ketosis, especially in children receiving concomitant valproate.^60^ However, most centers do not automatically start carnitine in all children. At this time, there is no evidence for the use of exogenous ketone esters or salts along with KDT for epilepsy.

## 8 MAINTENANCE OF KETOGENIC DIET THERAPY

After KDT is started, children should be seen back in clinic at 1, 3, 6, 9, and 12 months, with contact in between for questions.^1^ (*INFOGRAPHIC*) At most visits, laboratories are obtained including complete blood count, comprehensive metabolic profile, fasting lipid profile, antiseizure medication levels, and total and free carnitine. Additional laboratories may include Vitamin D levels, selenium, zinc, and serum beta‐hydroxybutyrate. At each visit, the family will meet with a neurologist and dietitian to discuss how KDT is progressing. Children will have their height and weight checked and plotted on growth curves. Most children respond to KDT within a few weeks, but typically KDT continuation is discussed at 3–6 months.

At home, parents will provide the ketogenic foods as prescribed and communicate with the dietitian for advice and adjust calories as necessary. Ketones are usually checked several times a week, either by urine acetoacetate strips or blood beta‐hydroxybutyrate (home meters). Parents are encouraged to contact the KDT with increased seizures, acidosis, over‐ketosis, signs of kidney stones, or weight loss.

## 9 ADVERSE EFFECTS OF THE KETOGENIC DIET

Since the creation of KDT, there has been concern about potentially harmful adverse effects associated with its use. As a result, there has been a large amount of research focused on both short and long‐term adverse effects, in particular within the pediatric population starting this medication for epilepsy. It is important for neurologists to understand these side effects, which unlike with antiseizure medications are manageable and should not usually be a reason for KDT to be discontinued. Adverse effects can be thought of as threefold—short term, long term, and laboratory derangements.

In the immediate period of starting ketogenic diet, most common adverse effects are constipation, abdominal pain, and vomiting. Much of this has to do with the composition of KDT. Given the need for low carbohydrate content, KDT has lower fiber and higher fat content, both of which can lead to generalized abdominal discomfort and constipation. Literature suggests that this is self‐limiting, but providers should manage symptoms accordingly with appropriate bowel regimen.^61^ Of note, abdominal pain should be re‐evaluated if it arises in the subacute to late phase of KDT due to other potential etiologies unrelated to constipation. There is a known risk of renal calculus formation, which is substantially reduced by use of sodium or potassium citrate but new abdominal pain should prompt broad differential.^59^ Aside from renal calculi, abdominal pain may also reflect pancreatitis which has been seen in a small cohort of patients on KDT.^62^ This is most common with patients already on ASMs with this side effect (i.e., valproic acid) or hypertriglyceridemia and providers should use their clinical judgment. In the acute period, in particularly after fasting to achieve ketosis, patients can also experience hypoglycemia. This has not been associated with poor outcomes long term but it does inform why KDT initiation is so often done with an inpatient admission—both for education and for monitoring of early adverse effects.^62^

Second to gastrointestinal complaints, the most common adverse effects seen with KDT are potential laboratory derangements. Most common effects include mild acidosis, hyperlipidemia, and hypoglycemia (mentioned earlier). Acidosis may occur in some patients and does not suggest increased clinical risk in most patients on KDT. Regarding lipid profiles, the highest risk for abnormal lipid profiles is within the first 6–12 months of KDT initiation. While this is true, these levels have been shown to normalize over time and at this time do not demonstrate higher risk of atherosclerotic change.^1^ There are a number of individual case reports which report vitamin and mineral deficiencies, but the most common and most likely to lead to clinical change are vitamin D and calcium. In patients who develop osteopenia or pathologic fractures on KDT, evaluation for serum levels should be initiated.^1^

If a patient is tolerating KDT well, they may be on it for up to several years depending on indication. With such prolonged exposure, there is potential for long‐term adverse effects in particular on growth—a common concern from families considering this treatment. Growth curves should certainly be monitored both by a patient's primary care provider and by their neurologist, but upon on review of literature it remains unclear what the impact of KDT alone is on growth with one study citing 9% of patients with growth one standard deviation below normal.^63^ In a small portion of patients, osteopenia has been noted which may impact bone health and growth, but this varies by age.^64^ Impact on growth seems most prominent in younger populations so should be a particular consideration in those patients starting ketogenic diet in infancy or even in the neonatal intensive care unit. While some studies have described “slowed growth” in patients on KDT, this is typically not pronounced enough that patients need to discontinue the diet.^63^

Despite this list of potential adverse effects, all are manageable with appropriate consideration by a neurologist. Serial laboratory monitoring is effective at identifying laboratory derangements early on. Renal stone risk can be mitigated by avoiding medications which independently increase risk such as carbonic anhydrase inhibitors. Use of potassium citrate can decrease renal stone risk in addition to reducing acidosis. Hyperlipidemia is screened prior to initiation and during KDT. In patients with known risk of osteopenia, vitamin D supplementation and endocrinology following can be important. Finally, gastrointestinal side effects are well managed by use of medications for constipation and gastroesophageal reflux.

## 10 DISCONTINUATION OF KETOGENIC DIET THERAPY

Length of therapy for KDT and the decision to discontinue are generally tailored by a patient and their particular situation. This can vary widely when we compare the different uses of KD, whether that be epilepsy refractory to several ASMs, infantile spasms, specific genetic disorders, or emergent use in FIRES/NORSE. Each of these etiologies has an individualized approach to discontinuation of therapy.

A general approach toward using KDT for epilepsy is to trial for at least 3 months to assess for response, as noted in recent consensus statement.^1^ In some cases, there can be an increase in seizure frequency, at which point immediate discontinuation should be considered. In the more typical cohort of patients with refractory epilepsy who have seizure reduction with KDT, the 2‐year mark is an appropriate time to consider discontinuation. Discontinuation considers a number of variables and is a balance of seizure etiology and frequency as well as side effects and impacts on growth as noted earlier. Because of this, there are a number of situations where it is appropriate to consider early discontinuation of therapy in particular for patients who are not tolerating these known adverse effects. This model mirrors the same consideration with ASMs—often evaluating tolerance and seizure burden at the 2‐year mark prior to weaning off.

There are multiple situations in which both prolonged and shortened courses of KDT can be more beneficial for patients. These should be considered independently of the 2‐year trial period as discussed prior to this. Infantile epileptic spasms syndrome (IESS) is a particular diagnosis in which a shorter course of treatment can be considered. In this population of medically refractory IESS, there is literature to suggest that if seizure freedom is achieved while on KDT, discontinuation at 6 months is equivalent to the classic 2‐year model.^65^, ^66^ This suggests that there is no benefit to seizure control with prolonged KDT >1 year in these patients and allows us to reduce unnecessary prolonged ketosis for this patients.

In cases of NORSE, FIRES, or super‐refractory status epilepticus, KDT is often used to break the status epilepticus, but the duration of KDT after this occurs is less clear. This is considered on a case by case basis and determined by responsiveness to treatment, but typically is 6 months, unless ongoing occasional seizures are problematic and seem KDT responsive.

Prolonged use of KDT (past 2 years) can be controversial given known side effects and impacts on growth and development. There are situations though where this prolonged use may be important for neurodevelopment and cessation of seizures. In Glut‐1 Deficiency Syndrome, patients are dependent on the alternative energy source provided by KDT. This allows for seizure control and avoids consequences of this disorder which can include developmental delay and movement disorders. While restrictive, KDT is the most effective for managing this syndrome for life.^67^ Pyruvate Dehydrogenase deficiency similarly demonstrates a chronic and positive response to prolonged use of KDT.^42^ It is therefore currently recommended for these conditions to stay on KDT as long as possible, possibly for life.^1^

There is published evidence regarding KDT discontinuation, should that be considered.^68^ Children with multiple ASM needs and hard to control seizures should be weaned off KDT carefully and slowly, with close monitoring for increased seizures during this period. To achieve careful removal of ketosis, the most effective way to wean off KDT is to reduce ratio by 1:1 every 1–4 weeks.^1^ This gradual change should allow providers and parents to assess for re‐emergence of seizures prior to complete discontinuation. The risk of recurrence with discontinuation is higher in patients with epileptiform spikes on EEG or focal abnormalities on MRI.^69^ It may be prudent to review MRI and consider an EEG prior to discontinuation, again similar to weaning off ASMs.^69^ In some cases as suggested already, prolonged use of KDT may outweigh the risk of discontinuation. In those patients who did not show clinical improvement while on KDT, the process of discontinuation can be much quicker over the course of days as there is no perceived risk of quickly stopping ketosis.^68^

## 11 CAN THE DIET BE RETRIED?

With the advent of adult epilepsy diet centers and the unfortunate reality of severe, refractory epilepsy that is not surgically amenable, it is a common question among epileptologists if KDT can be reattempted years later. A single study evaluated this scenario and included 26 subjects from two epilepsy centers, with the second KDT trial a mean of 6 years later.^70^ The results tended to be similar within each individual; success with the first trial was later seen with the reattempt, and vice versa. Overall, 50% of children responded to the second trial, which was less than the first trial (77%), but still similar to overall KDT responses.^70^ Therefore, these results suggest reattempting a previously successful KDT years later may be valuable.

## 12 SUMMARY

Ketogenic diet therapy is a valuable nonpharmacologic treatment of refractory epilepsy in children and adults. Significant evidence exists to guide initiation of the diet as well as choice of five specific diets to choose from. In addition, neurologists have identified several indications in which KDT is highly effective, even perhaps as a first‐line therapy. Side effects exist, are typically gastrointestinal, growth or dyslipidemias, and rarely require KDT discontinuation when they occur. Continued use of KDT over the next century will identify mechanisms of action and further improve efficacy and reduce adverse effects of this highly useful treatment.

## CONFLICT OF INTEREST STATEMENT

Eric Kossoff has served as a paid consultant for Simply Good Foods Inc., Nutricia, Inc., Bloom Science, Cerecin, and LivaNova, and has received royalties from Springer Medical Publishing, UpToDate and the Oxford University Press. None of the other authors have disclosures to report.

**Test yourself**

1. Which of the following is not a ketogenic diet therapy?
  - Modified Atkins Diet
  - Classic ketogenic diet
  - MCT diet
  - Gluten‐free diet
2. Which of the following is currently not an indication for KDT?
  - Absence epilepsy
  - Glut‐1 deficiency syndrome
  - Dravet syndrome
  - Epilepsy with myoclonic‐atonic seizures
3. Which is not an appropriate method of starting the diet?
  - Inpatient with a fasting period
  - Inpatient without a fasting period
  - Outpatient without a fasting period over 1–2 months
  - Outpatient with a fasting period over 24 h
4. Which of the following side effects are occasionally seen with KDT?
  - Kidney stones
  - Constipation
  - Gastroesophageal reflux
  - Bone fractures
  - All of the above
5. Kidney stones may be prevented by using this supplement:
  - Zinc
  - Potassium citrate
  - Atorvastatin
  - Selenium
6. Which laboratories are NOT universally obtained while on KDT?
  - Total and free carnitine
  - Antiseizure drug levels
  - Selenium levels
  - Comprehensive metabolic panel
7. Aspects of a ketogenic diet follow‐up clinic that are addressed include:
  - Seizure control
  - Height, weight and overall growth and tolerability
  - Antiseizure medication adjustment
  - All of the above
8. Which of the following is a contraindication for KDT?
  - Primary carnitine deficiency
  - Infantile spasms
  - Lack of a gastrostomy tube
  - Pyruvate dehydrogenase deficiency
9. The average rate of response (>50% seizure reduction) in children on KDT is:
  - 20%
  - 40%
  - 50%
  - 80%
10. The ideal duration of a wean off of the KDT is:
  - Over 2 weeks
  - Immediate stop
  - Over 6 months
  - Up to the individual patient and KDT center

*Answers may be found in the* supporting information.

## Supporting information


Data S1.

Data S2.

Data S3.
