# Supplementary material for: Ketogenic diet therapy for the treatment of pediatric epilepsy
Source: Epileptic Disord. 2024 Dec 12;27(2):144–55. doi: 10.1002/epd2.20320 (PMC12065128; doi:10.1002/epd2.20320)
Supplement: Supplementary file 1 — Data S1. [file EPD2-27-144-s002.docx]

**TEST YOURSELF**

**Answers:**

1. D

2. A

3. D

4. E

5. B

6. C

7. D

8. A

9. C

10. D
