# Supplementary material for: Ketogenic diet therapy for the treatment of pediatric epilepsy
Source: Epileptic Disord. 2024 Dec 12;27(2):144–55. doi: 10.1002/epd2.20320 (PMC12065128; doi:10.1002/epd2.20320)
Supplement: Supplementary file 2 — Data S2. [file EPD2-27-144-s001.pptx]

## Slide 1
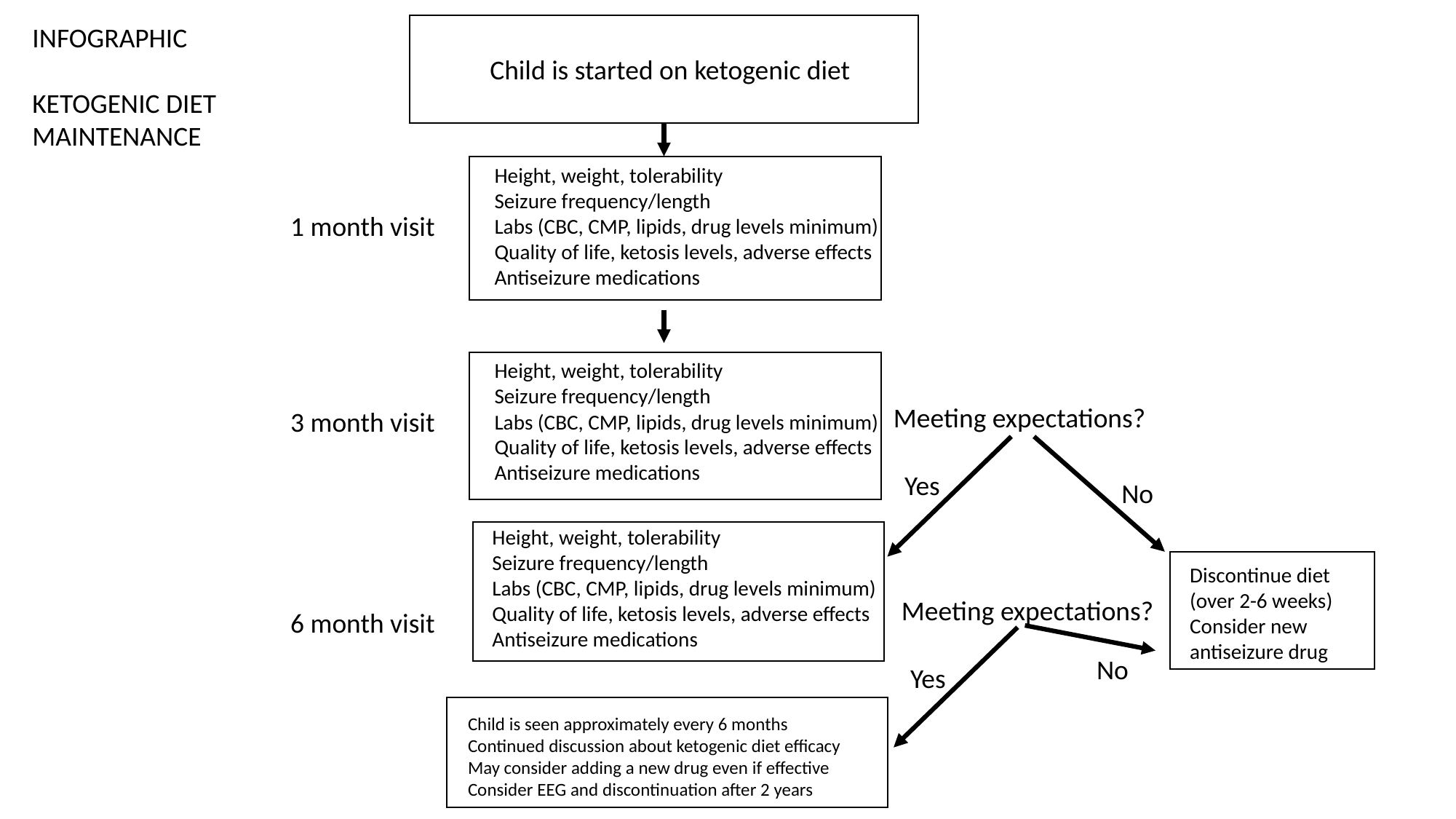

INFOGRAPHIC
KETOGENIC DIET MAINTENANCE
Child is started on ketogenic diet
Height, weight, tolerability
Seizure frequency/length
Labs (CBC, CMP, lipids, drug levels minimum)
Quality of life, ketosis levels, adverse effects
Antiseizure medications
1 month visit
Height, weight, tolerability
Seizure frequency/length
Labs (CBC, CMP, lipids, drug levels minimum)
Quality of life, ketosis levels, adverse effects
Antiseizure medications
Meeting expectations?
3 month visit
Yes
No
Height, weight, tolerability
Seizure frequency/length
Labs (CBC, CMP, lipids, drug levels minimum)
Quality of life, ketosis levels, adverse effects
Antiseizure medications
Discontinue diet (over 2-6 weeks)
Consider new antiseizure drug
Meeting expectations?
6 month visit
No
Yes
Child is seen approximately every 6 months
Continued discussion about ketogenic diet efficacy
May consider adding a new drug even if effective
Consider EEG and discontinuation after 2 years
