# Supplementary material for: Ketogenic diet therapy for the treatment of pediatric epilepsy
Source: Epileptic Disord. 2024 Dec 12;27(2):144–55. doi: 10.1002/epd2.20320 (PMC12065128; doi:10.1002/epd2.20320)
Supplement: Supplementary file 3 — Data S3. [file EPD2-27-144-s003.docx]

**Case Studies**

For *Ketogenic Diet Therapy for the Treatment of Pediatric Epilepsy*

1. A 16-month old girl is referred for ketogenic diet therapy for her refractory idiopathic infantile spasms. She is gastrostomy tube fed and her EEG continues to show modified hypsarrhythmia. Past medications tried include prednisolone, vigabatrin, topiramate, valproate, cannabadiol, and pyridoxine. As she is formula fed and has infantile spasms, she is identified as an excellent potential candidate. She is admitted and started on a 4:1 ratio ketogenic diet over 3 days without a fasting period and goes into moderate ketosis. Her concurrent topiramate is left unchanged and she is seen back in clinic after one month. Spasms are reduced by 50% and topiramate is slowly weaned to discontinuation. At the 3 month visit, her total cholesterol is >400 mg/dL and she is not growing well, so the ratio is lowered to 3:1. By 1 year of age, she is seizure-free. Her EEG shows occasional right temporal sharp waves, so levetiracetam is started, and the ketogenic diet is weaned gradually over 2 months back to a regular formula. She continues to be seizure-free 1 year later.
2. A 17-year-old boy is referred for ketogenic diet therapy for refractory focal epilepsy due to a left frontal cortical malformation. Seizures are occurring daily despite ongoing treatment with valproate, oxcarbazepine, and lacosamide. He had tried the classic ketogenic diet at another institution from ages 5-7 years with a 90% reduction in seizures, but never achieved seizure freedom and it was discontinued without resultant worsening. Now 10 years later, the family (and patient) are interested in re-trying ketogenic diet therapy rather than epilepsy surgery. He is started as an outpatient on a modified Atkins diet, restricting carbohydrates to 20 grams/day and encouraging high fat foods. After 1 month, he is in large ketosis, enjoys the foods, and has had a similar 90% reduction in seizures, with seizures occurring every other week. After 6 months on diet therapy, despite the improvement, he reports that the diet has become too restrictive for him and he wishes to stop. Seizures increase back to daily over the next 2 weeks and he is referred for epilepsy surgery consideration.
